# Resilience and Controllability of Dynamic Collective Behaviors

**DOI:** 10.1371/journal.pone.0082578

**Published:** 2013-12-17

**Authors:** Mohammad Komareji, Roland Bouffanais

**Affiliations:** Singapore University of Technology and Design, Singapore, Singapore; Semmelweis University, Hungary

## Abstract

The network paradigm is used to gain insight into the structural root causes of the resilience of consensus in dynamic collective behaviors, and to analyze the controllability of the swarm dynamics. Here we devise the dynamic signaling network which is the information transfer channel underpinning the swarm dynamics of the directed interagent connectivity based on a topological neighborhood of interactions. The study of the connectedness of the swarm signaling network reveals the profound relationship between group size and number of interacting neighbors, which is found to be in good agreement with field observations on flock of starlings [Ballerini *et al.* (2008) Proc. Natl. Acad. Sci. USA, 105: 1232]. Using a dynamical model, we generate dynamic collective behaviors enabling us to uncover that the swarm signaling network is a homogeneous clustered small-world network, thus facilitating emergent outcomes if connectedness is maintained. Resilience of the emergent consensus is tested by introducing exogenous environmental noise, which ultimately stresses how deeply intertwined are the swarm dynamics in the physical and network spaces. The availability of the signaling network allows us to analytically establish for the first time the number of driver agents necessary to fully control the swarm dynamics.

## Introduction

In an animal group, if each individual contributes independently to a given collective goal or objective, the resulting group behavior follows some sort of normal distribution pattern. On the contrary, if animals work collectively with a certain level of local interaction or communication, the output of their acts is more than the sum of each individual's act [1]. The emergent behavior is thus characterized by some signatures in the structural properties of the network underpinning their cooperative behavior [1]–[6]. Moreover, the global outcome of their local interactions heavily depends on each individual's initial conditions [7], [8]. For example the velocity of a flock of birds was found to be a function of each bird's initial velocity[9]. The emergence of spatiotemporal order at the group level has been observed in many biological systems [10]—insect colonies, fish schooling, bird flocking, amoebae aggregating, bacteria swarming, in many human activities [11], [12]—pedestrian and automobile traffic, and in the artificial world with robotic swarm systems [13].

Sumpter [14] argues that the key to understanding collective behaviors—and more broadly the concept of self-organization—lies in identifying the principles of the behavioral algorithms followed by individual animals and how information flows between the animals. That is what physicists, biologists and engineers have been trying to achieve through Lagrangian modeling of animals' collective behaviors as attested by the significant body of literature dealing with this specific issue [1], [9], [15]–[21]. Lagrangian swarming models are essentially built upon rules extended from some or all of the original Reynolds rules [15]—Cohesion: moving towards the average position of local flockmates; Alignment: steering towards the average heading of local flockmates; Separation: avoiding crowding local flockmates.

Vicsek *et al.* [16] introduced a simple discrete-time model of self-propelled particles with biologically motivated interactions. Particles in that model move in a plane with constant speed while aligning, at each time step, their velocity direction with their neighbors' average direction of motion. Jadbabaie *et al.* [17] provided the mathematical analysis and proof of convergence for Vicsek's model. Couzin *et al.* [18] developed a discrete model meant to consider leadership and decision-making issues in animal groups. In Couzin's model, at each time step, agents outside a given repulsion zone follow the desired direction of travel by two acts: first by moving towards the centroid of near neighbors, and second by getting aligned with the velocity direction of agents in the local interaction range. Olfati-Saber [9] introduced a flocking model based on a behavioral algorithm embodying an extended form of the Reynolds rules. Olfati-Saber's model is intrinsically continuous and has the interesting and appealing ability of representing flock characteristics such as rendezvous in space and obstacle avoidance. The Cucker–Smale flocking model [20] assumes birds adjust their velocity through applying a local linear consensus protocol which adds to the velocity a weighted average of the differences of its velocity with those of the other birds. The entire flock can therefore be represented by a complete weighted undirected graph whose weights are a function of distance between every two individual birds or nodes. The Cucker–Smale model can be either continuous or discrete. An extension of that model that guarantees the collision avoidance property can be found in Ref. [21].

Another approach toward the study of collective behavior is based on an analogy with the emergence of coherent behavior within a system of coupled oscillators achieving synchronization. Watts and Strogatz [22] studied the synchronization properties of real-world networks, while Lago-Fernández *et al.* [23] proved that clustering improves synchronization. Small-world systems corresponding to identical oscillators with linear coupling were studied by Barahona and Percora [24], while Nishikawa *et al.* [25] revealed that scale-free networks are more difficult to synchronize compared to homogeneous networks. A comprehensive application of this approach is given by Raley *et al.* [19] with a particular focus on how a network of coupled oscillators can be used to model the collective behavior of animals, with a special emphasis on fish schooling. This continuous model supposes particles can change their velocity heading but are unable to speed up or slow down. More information on problems of synchronization involving complex networks can be found in Ref. [26].

Despite these numerous efforts in developing continuous and discrete models, very little insight has been gained into the structure and dynamics of the information channel, which controls how information flows throughout the swarm [14]. Indeed, the vast majority of dynamical models reported in the literature are primarily focused on devising refined behavioral algorithms. The importance of deepening our understanding of this purely decentralized architecture flow among system's components can be readily acknowledged by recent discoveries of similar structures governing the very mechanisms underlying social self-organization [10].

In this paper, we bring together notions from ecology, network theory, information theory, control theory, and agent-based modeling to establish and comprehend the intricate relationship between the properties of the information transfer channel—referred to as the swarm signaling network in the sequel—and the dynamics of emergent collective behaviors based on local interactions and decentralized control. Particular emphasis is placed on gaining insight into: (i) what structurally makes swarming behaviors resilient or robust, and (ii) how controllable the swarm can be. To this aim, we explicitly define and construct the signaling network underpinning the group's interactions that represents connections between all group members in the physical space. This signaling network, channeling the flow of information between agents, has a unique dynamics which is intimately connected to the dynamics of the group members in the physical space. More specifically, we show that the group's dynamic signaling network is composed of directed links locally defined by a specific topological neighborhood of interactions for each and every agent. The study of the connectedness of the swarm signaling network allows us to uncover the pivotal relationship between swarm size and number of neighbors in the topological neighborhood of interactions, which proves to be in very good agreement with empirical observations obtained from flocks of birds. Using a dynamical model epitomizing our general framework, we analyze swarming behaviors by thoroughly characterizing the dynamics and structure of the signaling network. A profound connection between swarm dynamics in the physical space and dynamics in the signaling network space is uncovered. We find that swarm signaling networks are homogeneous and clustered small-world networks—known to be prone to yielding large-scale synchronization and emergence—even in the presence of environmental noise. Subsequently, the resilience or robustness of the collective emergent behavior is tested by adding exogenous noise in the environment. Depending on the number of neighbors considered, using the *k*-nearest neighbor approach, we show that consensus is achieved and maintained if the swarm signaling network remains as a single giant strongly connected component at almost all time. Finally, our analysis of the controllability of the swarm signaling network enabled us to establish for the first time the analytical expression of the number of driver nodes in terms of the swarm size and showing an exponential decay with the number of nearest neighbors in the neighborhood of interaction.

## Results

### Connectedness of the signaling network

Within our modeling framework (Methods section), the dynamic swarm signaling network (SSN) is explicitly accessible and one may ponder over the details of the relationship between connectedness of this network and emergent collective behaviors through local synchronization. Here, we propose to bridge the gap between two vastly different representations of the dynamics of our complex adaptive system. On the one hand, we have the prevalent canonical representation in the physical space—e.g. kinematic tracking of group members—and, on the other hand, the SSN approach in the ‘network space’.

**Figure 1:**
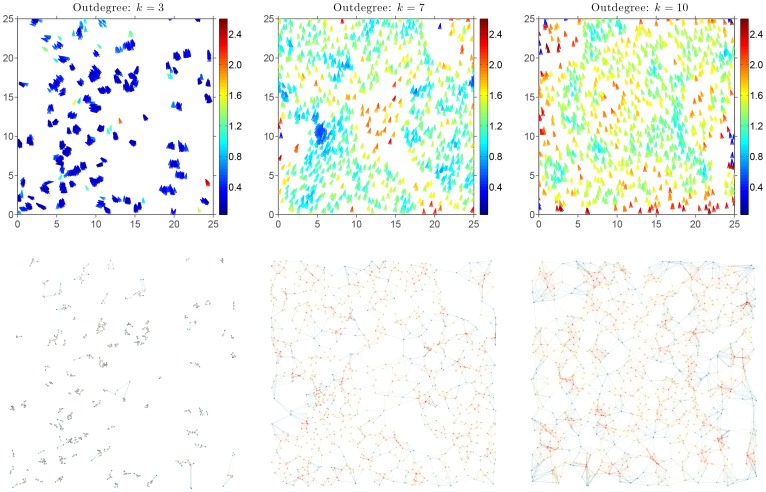
At a given instant, in a quasi-steady-state regime, velocity directions 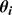 of 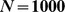 agents are displayed in the physical space (top row) and the associated SSN in the network space (bottom row) for three different values of the outdegree. 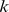
: Left column: outdegree 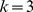; Center column: outdegree 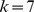; Right column: outdegree 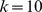. Top row: the actual velocity of an agent is indicated by a small arrow which color is mapped onto the size of the radius of the topological neighborhood of interactions. The vertical colormap is identical for all values of *k*, and the size of radius is expressed with the same spatial units as the square domain 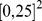. Roughly, a blue arrow corresponds to an agent with a fairly small topological neighborhood of interactions, while, on the contrary, a red arrow indicates a large topological neighborhood of interactions. Bottom row: instantaneous SSN associated with the physical distribution of agents shown in the top row. The network nodes are exactly located at the agents' physical locations. The directed links are colored according to the value of the indegree 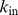 of the source node, also colored, from which they are originating. A linear colormap ranging from blue to red is used with three different indegree intervals: 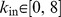 for 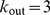, 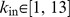 for 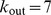 and 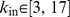 for 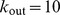. The results correspond to the time step 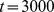 nondimensional time units, which according to the results in Fig. 9, is part of a quasi steady state. The noise level is fixed and set to 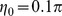 rad.

In the physical space, the emergent outcome appears before one's eyes (Fig. 1 top row). Reaching local synchronization is a key factor in forming a group and maintaining its emergent behavior, otherwise the group will split apart unless a consensus is reached again. Furthermore, consensus decisions bring along enhancement of decision accuracy compared with lone individuals and improvement in decision speed [27], [28]. For a group to self-organize, the union of the dynamically-evolving SSNs must have a spanning tree frequently enough [29]. Empirical evidences implicitly indicate the existence of a signaling channel between every two arbitrary agents in the swarm at any point in time. From the unique observations and findings of the STARFLAG project, Cavagna *et al.* [6] came up with this compelling statement: “The change in the behavioral state of one animal affects and is affected by that of all other animals in the group, no matter how large the group is”. Formally put, the SSN of the swarm is strongly connected at all time which is a much stronger condition than the one presented in Ref. [29].

**Figure 2:**
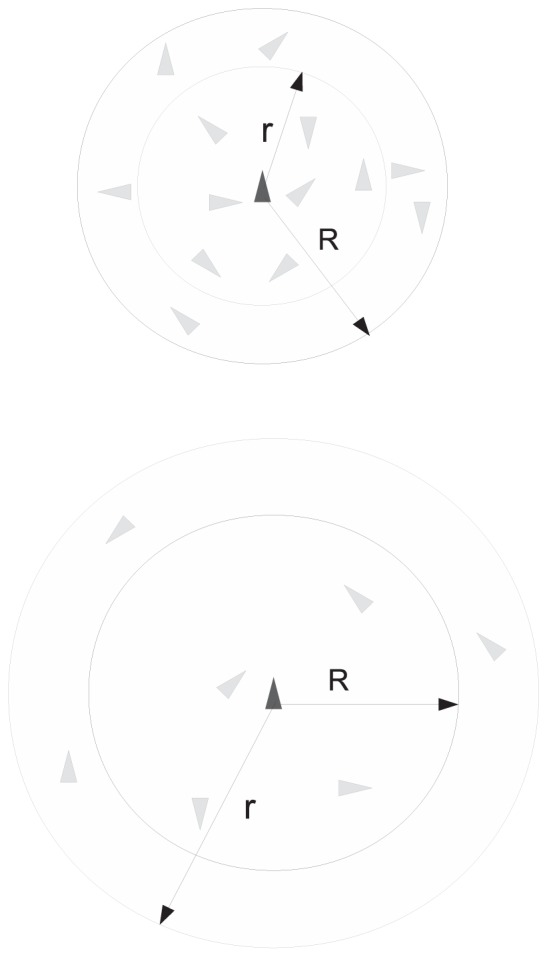
Schematics of metric (top) vs. topological (bottom) neighborhood of interactions. 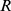
 is the radius of the metric neighborhood and *r* is the radius of the topological one based on the rule of *k*-nearest neighbors with 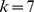. *R* is constant as it defines a metric zone around the agent while *r* changes in accordance with the distance between the agent and its *k*-th (here 7-th) nearest neighbor.

**Figure 3:**
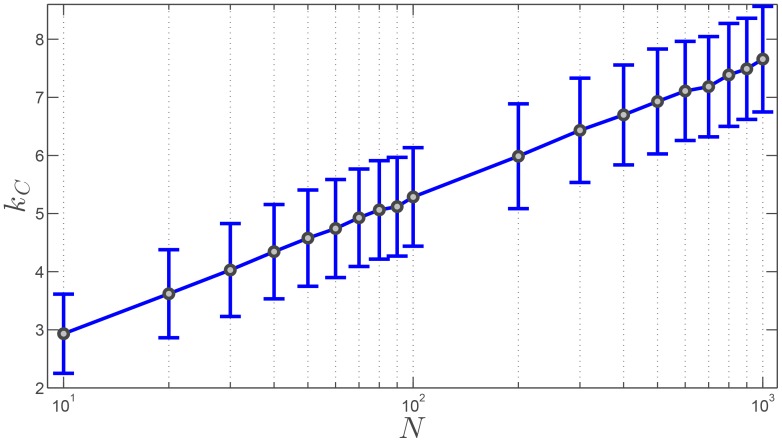
Critical value of the number of topological neighbors, 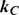, for which the connectedness of the network is guaranteed, as a function of the swarm size 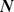, with 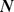 ranging from 10 to 1000. Grey dots represent the average value of 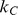 obtained from a statistical analysis comprising 1000 randomly generated *k*-nearest digraphs. The errorbars represent the associated standard deviations.

The very first characterization of the SSN pertains to its connectedness, which, in a 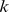-nearest graph representing the topological interactions (see Methods and Fig. 2 for an introduction to the differences between metric and topological neighborhoods), heavily depends on the value of the outdegree 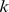 (Fig. 1 bottom row). The existence of a critical value, 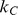, for the outdegree *k* such that for 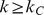 the *k*-nearest graph is connected, has never been proved. However, Balister *et al.* [30] proved the existence of 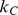 in the probabilistic sense. More specifically, they proved that for
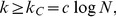
(1)where 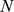 is the number of nodes—i.e. the number of agents in the group—the probability for any randomly-generated 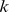-nearest graph to be connected tends to one. In Eq. (1), 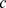 is a constant and the smallest value found so far is 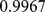 [30]. It is important keeping in mind that those mathematical results were obtained under the assumption that 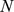 is large. When collective motion is considered, the number of agents considered ranges from dozens to a few thousands, and rarely more [1]. It is therefore important to assess numerically the validity of Eq. (1) for values of 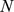 smaller than 1000. Figure 3 shows that even for small values of 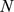, 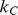 continues to scale linearly with 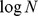 on average. Moreover, the average value of the coefficient 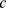 here is found equal to 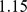
—this value tends to decrease with increasing values of 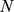, which is consistent with the value 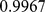 found in Ref. [30] for large values of 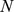.

Balister *et al.* [31] further expanded this result to the more conservative notion of *s*-connectivity. The SSN is said to be *s*-connected if it contains at least 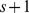 agents, and the removal of any 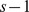 of its agents does not disconnect it. Obviously, the concept of 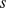-connectivity is instrumental to study the resilience of our dynamic SSN. Balister *et al.* [31] found that for 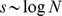, the critical outdegree 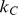 is asymptotically—i.e. for very large swarms—the same for the *s*-connectivity as for the regular connectivity. That is, as the outdegree *k* is increased, the SSN becomes *s*-connected very shortly after it becomes connected and the removal of a small number of its agent will not harm the swarm's connectivity. This property is consistent with a host of real-life observations on animal groups in nature [1], [32].

### Structure of the signaling network

#### Shortest connecting path

**Figure 4:**
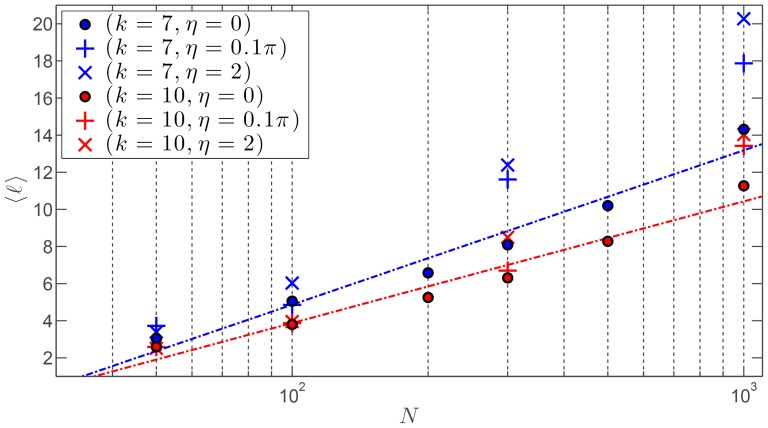
Average shortest connecting path vs. number of agents for the SSN. A log scale is used for the number of agents 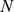. Two possible values of the outdegree are considered: 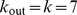 and 10. Three values of the noise level 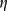 are considered: noiseless (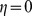), moderate (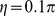 rad), high (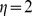 rad). The linear fitting in log scale is only shown for the noiseless case using dash-dotted lines.

Let us first consider the distance among agents in the swarm, and by distance here we mean the network distance between nodes representing the agents in the swarm network, and not the physical distance between agents in the physical space. Typically this distance is defined by the shortest connecting path, 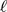, between any pair of agents. This metric is intimately related to the small-world effect, with which it is possible to go from one agent to any other in the swarm passing through a very small number of intermediate agents. To be more precise, the small-world property refers to networks in which the average shortest connecting path, 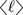, scales logarithmically, or more slowly, with the number of agents 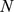. Figure 4 illustrates the average shortest connecting path 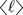 versus 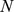 for two different outdegree values 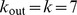 and 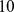 for our SSN, and for three vastly different noise levels—noiseless, moderate, and high. We chose those values for 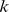 in order to ensure that the network remains connected for up to 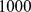 agents—the connectivity being necessary to compute the average shortest connecting path. Given the log scale on the 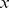-axis, our results clearly confirm that the SSN exhibits the small-world phenomenon for both values of the outdegree considered. Our empirical result is further supported by a very recent mathematical analysis by Alamgir & von Luxburg [33]. Not surprisingly, a higher outdegree shortens the shortest connecting path for all swarm sizes. On the contrary, 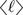 is lengthened when the swarm evolves in increasingly noisy environmental conditions, but the small-world property is conserved.

**Figure 5:**
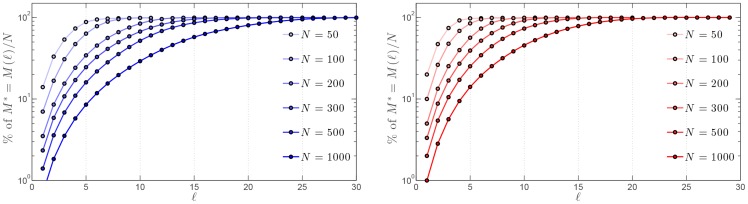
Normalized hop plot: 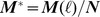 for the SSN. A log scale is used for the number of agents 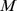 and various swarm sizes 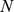 are considered. Two possible values of the outdegree are considered: Left: 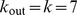; Right: 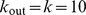. The noise level is fixed and set to 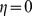.

The small-world property can be more thoroughly analyzed by inspecting the behavior of the quantity 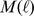 defined as the average number of agents within a network distance less than or equal to 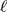 from any given agent [34]. The corresponding hop plot is shown in Fig. 5 for two values of the outdegree 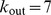 and 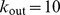. The exponential increase of 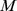 with 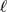 is yet another proof of the small-world character of the SSN.

#### Clustering coefficient

It is very interesting to observe that our swarm model (Methods section) based on the 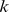-nearest neighbor topological neighborhood of interactions (TNI: Methods section and Fig. 2) generates a SSN showcasing the small-world effect. However, in many social and technological networks, the small-world effect is accompanied by a relatively high level of clustering. For instance, random networks also exhibit the small-world effect but possess an extremely low level of clustering.

**Figure 6:**
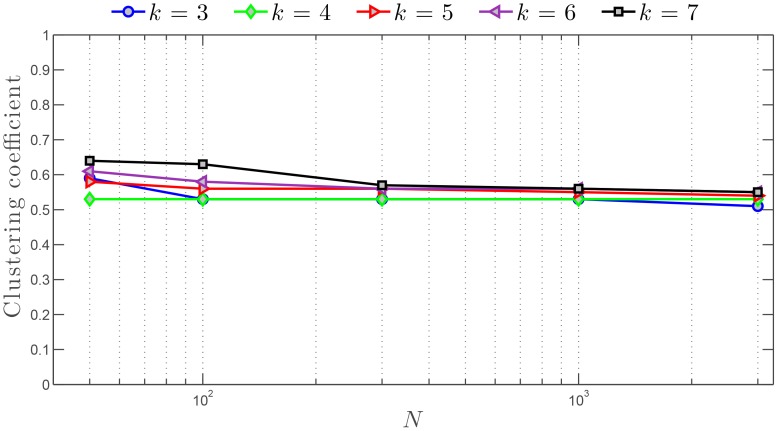
Clustering coefficient (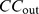) versus number of agents for the SSN. A log scale is used for the number of agents 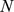. Different values of the outdegree are considered: 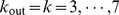. The noise level is fixed and set to 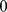.

The clustering coefficient, 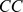, characterizes the local cohesiveness of networks [22] as well as the propensity to form clusters of interconnected elements. Given the directed nature of the SSN and the fact that neighbors are pointed at by outward edges, we consider the extended definition of the clustering coefficient 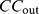 given in Ref. [35]. Thus, the average clustering coefficient of our 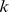-nearest neighbor graph can be calculated as follows [35]:
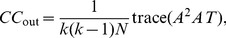
(2)where 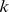, 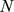, and 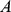 are the outdegree, the number of agents, and the adjacency matrix of the SSN, respectively [36]. Figure 6 shows the swarm's clustering coefficient as a function of the number of agents 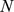 in the swarm, for several different values of the outdegree 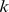, and in the absence of noise. These results highlight the rather high independence of the clustering coefficient with both the number of agents and the outdegree. We are therefore led to conclude that the SSN is intrinsically highly clustered unlike random networks. Interestingly, those measured levels of clustering are practically not affected by the presence of environmental noise—moderate (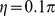 rad) and high (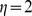 rad) noise levels were tested. We contend that the high level of clustering in the SSN may find its origins in the existence of clusters of agents in swarms, as commonly observed in nature [37].

#### Indegree distribution

We have established that the SSN is a clustered small-world network. To better understand its subtle structural organization, we now turn to the study of its statistical homogeneity. Homogeneous networks are characterized by fast-decaying degree distributions whereas heterogeneous networks produce long and heavy tails—such power laws are a well-known signature of scale-free networks [34].

**Figure 7:**
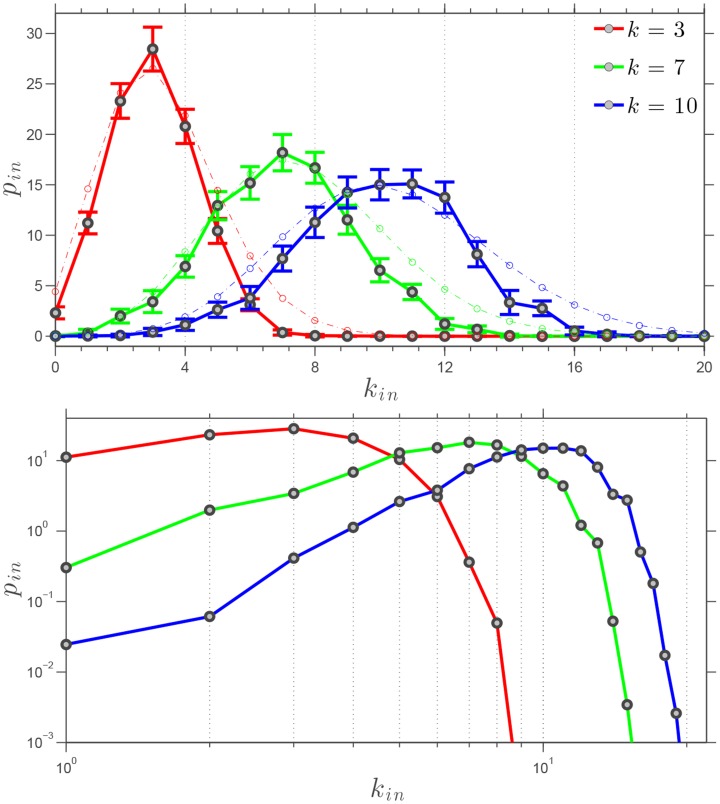
Indegree distribution 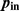 of agents in the SSN for several simulations of the swarming model with 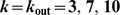 and different number of agents. 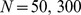
 and 1000; Top: linear scales with the exact values corresponding to the Poisson distributions for 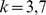 and 10 shown using thin dash-dotted lines, and Bottom: logarithmic scales. The average indegrees 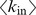 are 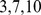 and their standard deviations 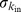 are approximately 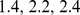, for 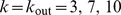 respectively. The noise level is fixed and set to 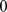.

The indegree, 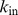, of an agent in the SSN is the number of directed edges pointing at it; a directed edge representing a neighboring agent using the information from the state of the agent that its edge is pointing at. The indegree distribution, 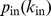, is the fraction of agents in the SSN having an indegree 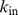. The average indegree distribution, 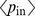, for our SSN is computed for three distinct values of the outdegree, 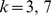 and 10. The averaging 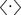 considered is a mixed conditional averaging based on a temporal averaging of the network configurations for 800 consecutive timesteps—with 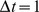
—repeated 8 times each, and that for three different values of the total number of agents: 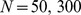 and 1000. It is important to note that our results show very little variation in the average indegree distributions for the three values of 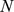 considered. The results are shown in Fig. 7, in which the errorbars represent the standard deviation to the average value found. The indegree distributions are peaked at 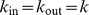 for the three values of the outdegree considered. More precisely, approximately half of the swarm agents have an indegree such that 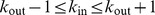. Furthermore, for 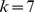 and 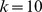, the indegree distribution is qualitatively symmetric about their maximum value obtained at 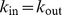. Based on the log-log plot of the indegree distribution in Fig. 7 (Bottom), it can be said that the indegree distributions clearly are Poissonian like, with 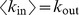 and with a variance increasing with 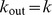. This is further verified by comparing the results with the actual Poisson distribution as shown in Fig. 6 (Top) with a relatively good qualitative agreement. Such Poissonian-like distributions are reminiscent of random networks and starkly differ from power laws characteristic of scale-free networks. Similarly to the clustering coefficient, measured indegree distributions are practically not affected by the presence of environmental noise—moderate (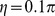 rad) and high (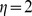 rad) noise levels were tested.

To further confirm the absence of an intrinsic characteristic scale for the SSN, we computed the heterogeneity parameter 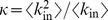. Homogeneous networks are known to have a 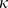 that scales with the indegree 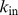 [34]. Table 1 shows the values of the reduced heterogeneity parameter 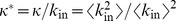 for 9 SSNs corresponding to three values of the outdegree 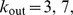 and 10, and for 3 different sizes of swarms corresponding to 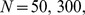 and 1000 agents. These results confirm the homogeneity of all our SSNs as 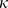 indeed scales with the indegree 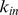, irrespective of the outdegree and swarm size. That allows us to conclude that our SSNs are homogeneous and clustered small-world networks.

**Table 1:** Reduced heterogeneity parameter 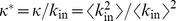 for 9 SSNs corresponding to 3 values of the outdegree 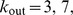 and 10, and for 3 different sizes of swarms corresponding to 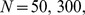 and 1000 agents.

*N*	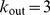	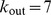	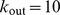
50	1.21	1.12	1.10
300	1.21	1.10	1.06
1000	1.31	1.09	1.08

### Resilience of the consensus

**Figure 8:**
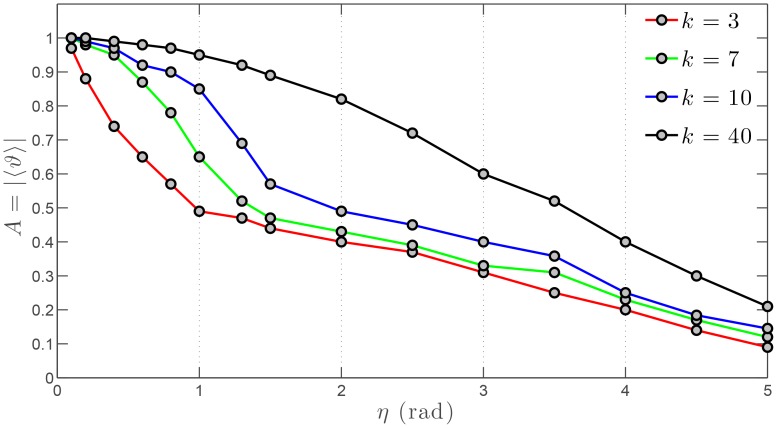
Alignment 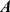 versus noise level 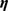 for a swarm comprised of 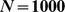 agents. Three values of the outdegree are considered: 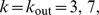 and 10.

The effects of noise on the dynamics of collective behaviors in the physical space is well known and has been thoroughly investigated in the case of a metric neighborhood [1], [16]. However, very little is known about those effects in the case of a TNI, and more importantly on the dynamics of the associated SSN. To this aim, we consider a swarm of 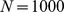 agents evenly distributed throughout the physical domain, subjected to periodic boundary conditions. Initially, all agents are heading North which globally yields an alignment of unity. Figure 8 shows the impact of noise on the alignment—i.e the consensus—of the swarm. In our framework, the alignment is used as a measure of the resilience of the ordered phase of the collective behavior to the effects of noise. As expected, the higher the noise level 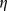, the lower the alignment. For relatively low noise levels 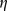, the decay of the alignment is faster for lower values of the outdegree 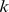. For higher values of 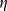, the decay of 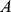 slows down and becomes almost the same for the four values of the outdegree considered.

**Figure 9:**
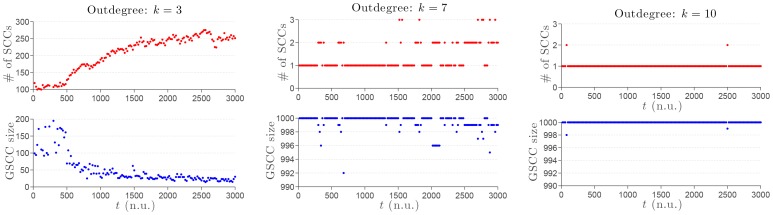
Dynamical properties of the GSCCs making the SSN. A dynamic range of 3000 nondimensional time units (n.u.) is considered with 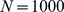 agents evenly distributed and all initially aligned with the North direction. The noise level is fixed and set to 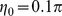 rad. Top row: total number of SCCs. Bottom row: size of the GSCC found in the SSN. Left column: outdegree 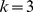; Center column: outdegree 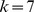; Right column: outdegree 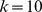.

The analysis of the SSN allows us to comprehend the above observations and trends. We now fix the noise level at 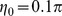, which falls right into the range where the alignment is significantly influenced by the outdegree. At the very beginning, prior to any interaction, the SSN is strongly connected for 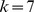 and 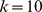 and it forms a single giant strongly connected component (GSCC) as is shown in Fig. 9 (top row). On the contrary, for 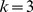 the SSN is composed of 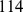 SCCs of very many different sizes: ranging from 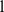 agent to 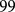 agents (Fig. 9, top row). Another informative quantity is the average neighborhood radius for the entire swarm—the neighborhood radius is given by the largest distance separating a given agent and its 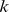 nearest neighbors. The initial average neighborhood radii are 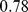, 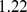 and 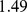 for 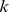 equals to 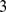, 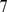 and 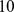 respectively. We then let this complex system evolve through local interactions of the agents and after a long-enough transient, the collection of agents yields vastly different emergent behaviors in both the physical and network spaces as shown in Fig. 1.

For the low outdegree 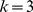, we observe a large number of clusters of locally-aligned agents; no large-scale emergent coherent alignment is achieved. This is clearly noticeable in both the physical and network spaces (Fig. 1, left column). The average TNI radius fell sharply from 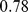 to 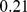 which is consistent with the physical clustering. Furthermore, the dynamics has amplified the fragmentation of the SSN, which, after the transient, contains 267 SCCs of much smaller sizes: ranging from 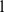 agent to 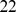 agents (Fig. 9, left column). Note that the number of SCCs for 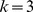 tends to reach an asymptotic plateau about the value 250 with very small-amplitude fluctuations after approximately 2000 nondimensional time units. We qualify this regime as quasi steady state. On the contrary, for both 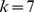 and 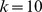, a large-scale coherent alignment is achieved while the distribution of agents is nonuniform but not as physically clustered as in the case 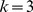. Those observations are corroborated by the fact that the SSN remains as a single giant strongly connected component—apart from very few agents splitting away from the “peloton” (Fig. 9, center and right columns)—with almost unchanged average TNI radii of 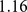 and 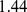 for 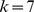 and 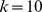 respectively. Furthermore, with a much larger value of the outdegree, 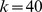, the swarm exhibits a higher level of resilience to noise with quite different variations of the alignment with the noise level as compared to other smaller values of 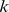 considered.

### Controllability of the signaling network

If one wishes to control the dynamics of collective behaviors—a goal of tremendous importance for both natural and artificial swarms, we now know that it is necessary identifying the swarm's architecture, in other words the SSN. From the engineering control viewpoint, such a dynamical system is said to be controllable if it can be driven from any initial state to any desired final state in finite time. Owing to the seminal work by Liu *et al.* [38], we know that it is first necessary to identify the set of agents that, if driven by different signals, can offer full control over the SSN. Liu *et al.* [38] developed the analytical tools to study the controllability of an arbitrary directed network allowing one to identify the set of driver agents. Specifically, they proved that we can gain full control over a directed network if and only if we directly control each unmatched node—a node is said to be matched if a link in the maximum matching points at it; otherwise it is unmatched— and there are directed paths from the input signals to all matched nodes.

The connectedness of the swarm signaling network is a sufficient condition for an agent within the swarm to affect and get affected by some if not all agents of the group. However, in many occasions, one or more agents need to be able to drive the swarm to a certain global state, and usually within finite time. This is better understood when considering two biological systems such as a flock of birds or a school of fish. For instance, evasive maneuvers triggered by predator or collision avoidance collective responses are induced by one or a few agents perceiving the threat and responding to it. These few agents effectively are driver agents in the abovedefined sense: they are able to control the entire swarm by bringing the other agents to swiftly respond to a threat that they are not directly detecting. It is worth adding that those driver agents do not possess any “super” power of any sort but they simply become drivers as they happened to have discerned the danger first; any other agent in the swarm could be driving the group as long as it is subjected to specific external cues which are not made available globally to the whole swarm. In summary, for a specific dynamic collective behavior to occur, connectedness and controllability of the SSN are necessary conditions.

A system's controllability is to a great extent encoded in the underlying degree distribution, 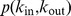. That is, the number of driver agents is determined mainly by the number of incoming and outgoing links each node of the SSN has, and is independent of where those links point at [38]. By construction the outdegree distribution of the SSN is a Dirac delta distribution, while we found that its indegree distribution very much resembles the one of a 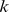-nearest random digraph. To allow for an analytical study of the controllability of the SSN, we therefore consider the following degree distributions:
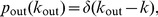
(3)

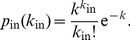
(4)


*Lemma.* The number of unmatched nodes of a graph having 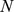 nodes and a constant outdegree such that 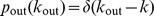, and an indegree distribution of Poisson type 
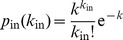
 is given by 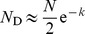, in the large 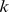 limit.

**Figure 10:**
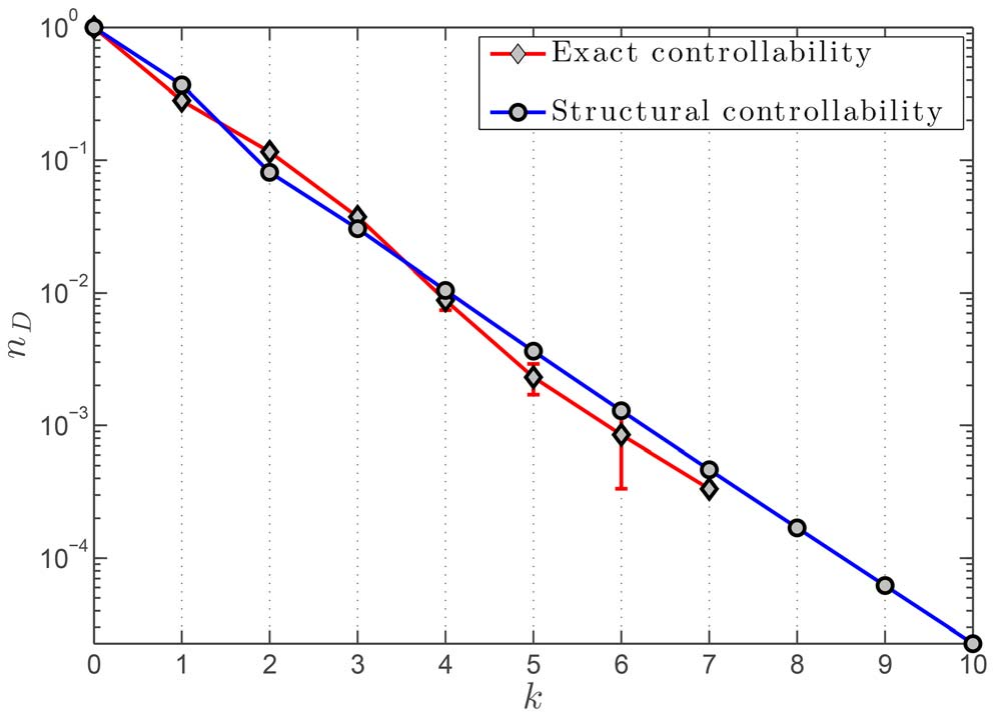
Density of driver agents, 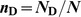, giving the proportion of agents necessary to control and drive a swarm of 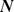 agents as a function of 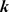, for a swarm dynamics with a topological neighborhood of interactions based on the 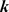 nearest neighbors. The exact controllability framework is the one by Yuan *et al.* [39], while the structural controllability framework was developed by Liu *et al.* [38]. Results using the exact controllability framework were obtained for 20 SSNs associated with a swarm of 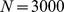 agents for each data point; beyond 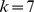, 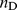 drops to zero and the values are hence not shown. The average density of driver nodes was calculated and the associated standard deviations are shown using the errorbars.

*Proof.* Following the approach developed by Liu *et al.* [38], the number of unmatched nodes, i.e. the minimum number of driver nodes 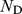 necessary to fully control the system, can be obtained from the following generating functions
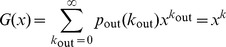
(5)

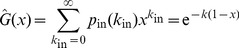
(6)

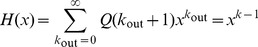
(7)

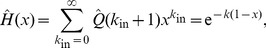
(8)where
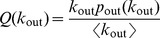
(9)

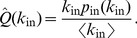
(10)The general expression for the number of driver nodes 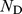 obtained by Liu *et al.* [38] is given by
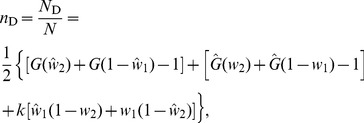
(11)where, in the SSN framework
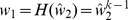
(12)

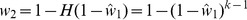
(13)

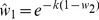
(14)

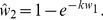
(15)When 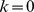, the agents are totally independent and 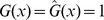. Hence, we trivially get 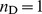 from Eq. (11), which simply means that we need to control 100% of the agents to control the dynamics of the swarm—this conclusion is consistent with the noninteracting dynamics of the group due to the choice of a 0-nearest neighborhood of interactions. We now turn to the other pathological case, 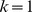, for which 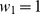, 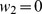, 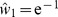, 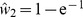, such that 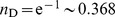. For 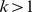, it is easy to check that 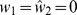 are the smallest roots for 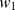 and 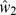 in the system of Eq. (12) and Eq. (15). Hence, the fraction of driver nodes simplifies to
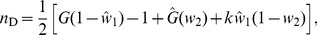
(16)or more explicitly
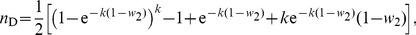
(17)in which *w*_2_ is solution of the self – consistent equation
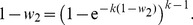
(18)With those results, 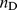 can easily be calculated and results are shown in Fig. 10. The asymptotic behavior of 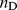 in the large 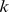 limit can easily be determined as 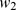 tends to 1. Hence, at the leading order
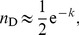
(19)which appears very clearly on the graph in Fig. 10 given the log scale on the 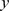-axis. This concludes the proof of the above Lemma.

It is important noting that within the structural controllability framework developed by Liu *et al.* [38], binary link weights such as those considered in the SSN (see Methods section and Eq. (23)) cannot be considered per se as they must be free independent parameters. This issue can readily be resolved by considering the more realistic case of non-binary weights accounting for the imperfections of the information transfer channels through which the agents interact. Alternatively, one may consider the exact controllability framework very recently developed by Yuan *et al.* [39], which offers a more universal tool to evaluate the controllability of any complex network. As is shown in Fig. 9, the results from both frameworks—structural controllability and exact controllability—are fully consistent.

The last question that should be answered regarding the above result on the number of driver nodes and the overall controllability of the SSN lies with the dynamic nature of the SSN. Since the SSN is intrinsically a switching network—at each instant a certain number of links are broken while the exact same number of edges are created due to the motion of the agents in the physical space—one can prove using Eq. (19) that it is controllable at each instant, assuming of course a high-enough value of *k*. If that is the case, it is known from control theory associated with dynamic multi-agent systems that the overall switching dynamical system is controllable [40], [41].

## Discussion

**Figure 11:**
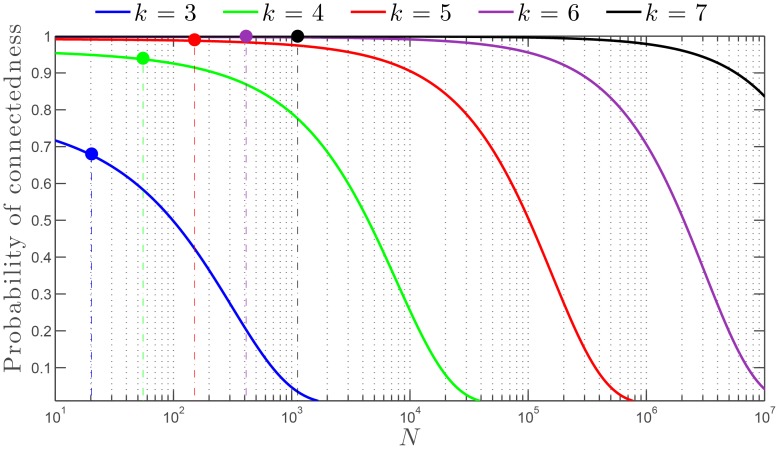
Probability of connectedness for the SSN vs. number of agents 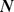 for different values of the number of nearest neighbors 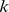. The SSN corresponds to a specific configuration of the swarm in which 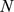 nodes are placed in a unit square independently through a uniform distribution. Then each node is connected to its 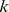 nearest neighbors to form the 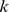-nearest graph. For each value of the outdegree 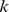, the maximum size of the swarm population 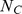
—given by 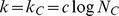 with 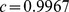 [30]— ensuring the connectedness of the SSN is represented by a colored dot with the associated vertical dashed line.

The study of the connectedness of the SSN allowed us to uncover the existence of a relationship between the swarm size, given the number *N* of agents, and the number *k* of nearest neighbors influencing any agent's behavior and dynamics. Indeed, the general results from graph theory applied to the study of the SSN connectedness take a particular significance in the context of dynamic collective behavior where the number of agents *N* may not necessarily be very large and the number of nearest neighbors, *k*, cannot possibly exceed at most 15 to 20 due to the intrinsic bandwidth limitations in signaling, sensing and internal information processing. To better appreciate these results, we present in Fig. 11 the dependence of the probability of connectedness of the SSN as a function of *N* for different values of *k*. Despite the uniform character of the distribution of agents in the swarm considered to establish Fig. 11, this figure reveals the profound relationship between connectedness of the swarm and the number of agents *N*, for different values of the outdegree *k*. This result was already suggested by Eq. (1). For the sake of explanation, let us consider a swarm comprised of 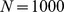 agents, which is a reasonable number for living animals [37]. Figure 10 shows that this swarm will remain connected at all time if 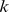 has at least a value of approximately 6 or 7. This result is in very good agreement with the experimental observations of Ballerini *et al.* [42] for flocks of starlings with approximately 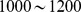 birds at maximum. Based on their thorough analysis of the dynamics of flocks, Ballerini *et al.* [42] claimed that each starling had a TNI made up of 6 to 7 other birds. Thus, our model leads to a more general rule of interaction in swarms: each agent interacts on average with a fixed number of neighbors irrespective of the distance, and that number of neighbors *k* depends on the swarm size *N*. By extension, for artificial swarms, which typically have a much smaller size—with say *N* being at most 100—our analysis enables us to conclude that 4 to 5 interacting neighbors are necessary to ensure the swarm's connectedness and effectiveness. Note that, this analysis based on Fig. 11 does not account for the dynamics of the SSN and more importantly for the ubiquitous presence of noise in the environment.

Beyond the connectedness of the SSN, we found for the first time the details of its structural properties revealing that, if connected, the SSN is a homogeneous and clustered small-world network even when considering the disruptive effects of noise on the inter-agent interactions. Hence, the swarm information transfer channel has a relatively high local cohesiveness and no intrinsic characteristic scale could be found in the indegree distribution. The small-world phenomenon could have been intuited through the mere observation of exceptionally fast responses of biological swarms to external cues, e.g. fish school evasive maneuver, collision avoidance, etc. The homogeneous character of the SSN could also have been intuited. Indeed, the difference in indegree distribution has vastly significant implications for the structure of the networks. For instance, the long tail of power-law distributions of the indegree is a clear signature of the existence of hubs in scale-free networks. Interestingly, even though our swarm network is not, per se, a random network—its dynamics is governed by a set of rules, including the 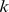-nearest neighbor rule—its indegree distribution is not able to reflect those differences with real random networks. Note that, this result is not surprising given that we are dealing with a collection of identical agents with a very minimal level of state properties; a power-law signature with the associated hub effect seems unthinkable in our context. However, we nonetheless observe that some specific agents do “attract” much more attention than others with indegrees of 15 and above (Fig. 7). Finally, it is interesting comparing the structural properties of the SSN based on a TNI with the ones for a signaling network based on a metric distance. Both interaction distances lead to similar levels of clustering and similar average shortest connecting paths. The central difference between the two groups of SSNs lies with the fact the topological SSN is a directed network while the metric SSN is undirected. As a direct consequence of that, the outdegree distributions of both types of SSNs are fundamentally different: the outdegree of the topological SSN is constant and equal to 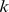, while the outdegree of the metric SSN is identical to the indegree distribution, which we found to be Poissonian-like.

A central point to always keep in mind is the fact that the SSN has a dynamics that is evolving hand in hand with the dynamics of the agents themselves. Hence, the connectedness and the structural properties of the SSN are in general not constant. Our analysis reveals this profound connection between, on the one hand, the dynamics of the collection of agents in the physical space and the structural properties of the SSN as well as its own dynamics, on the other hand. This comment is very elegantly epitomized by Fig. 1 which stresses the parallel between the structure of the swarm in the physical space and the associated SSNs for the three different values of the indegree considered, namely 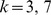, and 10. The instantaneous SSNs associated with the physical distribution of agents are shown in Fig. 1, bottom row. The network nodes are exactly located at the agents' physical locations, and the directed links are colored according to the value of the indegree, 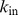, of the source node from which they are originating. For instance, we are able to visually correlate high values of the indegree 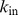 to small radii of the TNI. A better understanding of this observation would of course require a more thorough analysis which is beyond the scope of the present study. Another point has to be made about the connection between SSN structure and swarm dynamics in terms of consensus speed. Intuitively, one can easily imagine that a larger number of topological numbers *k* leads to faster consensus since the connectivity of the network underpinning the dynamics of the interacting swarming agents affects profoundly the consensus capability—in general, higher degree of connectivity yields higher rate of convergence to consensus [43]–[46]. This fact has very recently been proved exactly by Shang & Bouffanais [47]. However it is important to note that adding more edges by increasing the number of topological agents with whom one is interacting is feasible but only up to a certain extent as there is always a cost associated with information exchange and also due to inherent limits in terms of signaling mechanisms, sensory and cognitive capabilities—for instance, see Ref. [48] for such biological considerations with pigeons and Ref. [49] for SPPs having a limited view angle.

In our framework we considered the simplest topological model of all consisting in having the same number of nearest neighbors *k* for all agents. Obviously, this framework can be extended in many ways but one particular extension is worth mentioning: the case where *k* varies from agent to agent depending on some local parameters, e.g. the neighbors density of neighbors, the size of TNI radius, etc. Such a local adaptation of the value of the outdegree *k* clearly enforces a very specific outdegree distribution. Some very recent works on the controllability of complex networks [50], [51] allow to conclude that this would have a direct impact on the swarm controllability. Hence, this leads to the following intricate inverse problem of finding one or more distributions of *k* generating an optimal controllability of the swarm.

From the practical standpoint of designing artificial swarms, our knowledge of the properties and dynamics of the SSN, and their influence on the swarm dynamics is necessary but not sufficient. Gaining a better understanding of its controllability is paramount. Through Eq. (17) and Eq. (19), we have analytically established that the number of driver nodes decreases exponentially as the number of nearest neighbors increases. Note that for a metric-based SSN, the density of driver nodes is easily obtained as 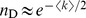 [38]. In addition, the value 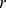 of the radius defining the metric neighborhood conditions the value of the mean degree 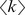. If one chooses a topological neighborhood such that 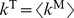
—where the superscript “T” refers to topological and “M” to metric—then the topological SSN can be said to be more controllable as 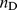 decreases faster with 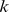 as compared to the metric case. Note that in the case of hierarchical group dynamics such as those reported by Nagy *et al.* [52], the signaling network has a well-defined tree structure. The controllability of such networks has been analytically established in Refs. [53], [54].

We can say that if the number of nearest neighbors reaches a value of 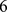 or 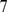
—for instance considering a flock of birds like those studied in the field by Ballerini *et al.* [42]—every agent not only affects and is affected by all other agents within the group, but more importantly, is capable of full control over all other agents. More generally, when a large swarm is considered its effectiveness and resilience entail the connectedness of the SSN. From Eq. (1), we can consider that the number of interacting neighbors is at minimum 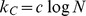, hence leading to 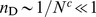 using Eq. (19). This result proves that ensuring the connectedness of large swarms automatically ensures its full controllability. However, it is possible that this interesting result ceases to be true for very small swarms. In summary, this ability to control the swarm is instrumental in situations where an agent—or even a few number of them—needs to play a leadership role in guiding the swarm either toward a certain destination or away from a potential danger. Note that this leadership role can be temporary or permanent.

## Methods

### General features of the model

Here, swarming refers to a circumstance in which multiple adaptive agents—be them living creatures or artificial ones—create a certain level of spatiotemporal order characterized by one or more macro-level properties. For the sake of clarity, we consider a collective of 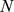 locally-interacting adaptive and identical individuals. Each individual agent 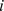, at any given instant 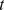, is assumed to be fully characterized by the state variable 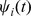. Such a generic state variable may represent widely different characteristics depending on the nature of the group considered: e.g. employed or unemployed forager state for honey bees, kinematic variables for fish in a school, birds in a flock or robots in an artificial swarm, space available for a pedestrian on a congested sidewalk, etc.

The nonlinear dynamics of each agent 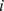 takes the general form
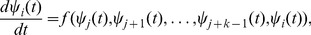
(20)that stresses the local nature of the interactions between agents since the subset 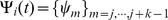 only includes a fraction 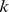 of the 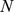 agents affecting the behavior of agent 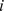. Note that the formalism of Eq. (20) does not capture the fact that the value of the 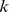 indices—from 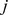 to 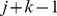 above—are actually *i*-dependent since they are defined by the belonging, or not, of an agent to the neighborhood of interaction of agent *i*. Moreover, these *k* indices may change over time due to the dynamical nature of the neighborhood of interactions, itself imposed by the dynamics of agent *i*. That means that in general, the makeup of 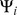 varies from individual to individual and changes with time. Specifically, it is entirely dependent on how the neighborhood of interactions—formally represented by 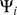
—is constructed which further defines the communication links between agents. The neighborhood of interactions is the cornerstone of the global SSN, and its intricate structural properties and dynamics have been studied below. Moreover, the values of each 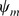 within 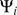 are made available to the internal control processing mechanism through the various sensory modalities defining multiple communication channels between group members—e.g. mechanical signaling through lateral line sensing and visual signaling are both involved in fish schooling [37]. The function 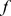 in Eq. (20) embodies the specifics of each individual's internal control processing mechanism. It is worth highlighting at this stage that complex collective dynamics can be achieved with simple 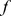 given the possibly nontrivial dynamics of 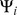 depending on the very nature of the neighborhood of interactions.

At this point, we make another general assumption consisting in imposing that any decision made by a group member is based on relative state values and not on absolute ones. If the state variable 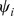 is a quantity that is frame dependent, such as the agent's velocity, the agent is solely able to appreciate an interacting neighbor's state with respect to its own. This argument may even hold for non-frame dependent state variables—e.g. pheromone levels in ant trails—and is easily reconcilable with the multiple gradient-based taxes observed in many biological systems [55]. Formally, this relative-state assumption reads
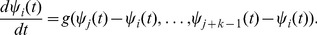
(21)The function 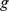 is referred to as a consensus protocol—intrinsically local by the nature of its inputs 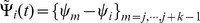
—if a steady-state can be reached and once it is reached, if the following relations hold: there exists a function 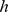 such that
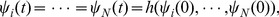
(22)where 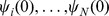 are agents' initial state conditions, e.g. agents' initial velocity directions in Ref. [43]. In simple words, the local synchronization protocol defines for each individual agent what Sumpter [14] calls the behavioral algorithm, also known as the internal information processing mechanism responsible for the behavioral's response to the sensed external information that is flowing in a decentralized way throughout the swarm.

### Topological neighborhood of interactions

We now aim at formalizing the key concept of neighborhood of interactions. From our introduction above, it appears clearly that 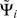 fundamentally depends on a series of factors which include: signaling mechanisms, sensory and cognitive capabilities. The signaling mechanisms are the different vehicles for the information to flow through the swarm's surrounding environment. The sensory capabilities are responsible for information acquisition from the surrounding environment to the internal agent domain. Within that domain, the internal information processing is taken care of by the cognitive capabilities. Even though the information chain has been clearly identified, we believe that accurately modeling each and every component is nonessential. Indeed, one and only one of those components will be the limiting factor and depending on the environmental conditions, that limiting factor may change; e.g. fish schooling from crystal-clear waters to murky ones [56]. Therefore, we consider a topological neighborhood of interactions (TNI) [57] whose physical relevance was discussed in Ref. [58].

The vast majority of models of collective animal behaviors found in the literature are based upon a metric neighborhood of interactions. In that specific class of models, the only thing that matters for an agent is the physical distance to neighboring agents. A typical example of an agent's metric neighborhood is the open ball interaction zone with radius 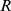 centered about the agent. The simplicity of the metric-based neighborhood approach is evident and that translates into a relative ease of computational implementation. However, it suffers from many limitations; for instance it cannot account for the cognitive limitations of agents evolving in very dense crowds [37].

European project named Starlings in Flight or STARFLAG has been one of the most recent and largest experiments in the human history carried out to analyze the collective behavior of birds [42]. By reconstructing the three-dimensional positions of individual birds in airborne flocks of a few thousand members, Ballerini *et al.* show that the interaction does not depend on the metric distance, as most current models and theories assume, but rather on the topological distance. They discovered that each bird interacts on average with a fixed number of neighbors (six to seven), rather than with all neighbors within a fixed metric distance. To the best of our knowledge, an explanation for this surprising empirical observation has yet to be given. Ballerini *et al.* [42] claim that interactions based on metric distance is unable to reproduce the density changes, typical of bird aggregations, because one would expect cohesion to be lost when mutual distances become too large compared with the interaction range. In addition, with social networks, the relevance of the topological distance between neighbors becomes apparent and it is believed that it could determine how populations move in, split up and form separate groups [59], [60]. For instance, guppies preferentially shoal with individuals of a similar size [61], and faster individuals are more likely to be found at the front of groups [62].

With a TNI, one has to be watchful for the possibility of the topological distance becoming too large so that the interaction or information exchange could not take place. In practice, that can potentially happen with very low density swarms or when some individual agents become widely separated from the swarm. In our numerical framework, the existence of periodic boundary conditions combined with a relatively high density of agents prevent such extreme case from happening. Still with a TNI, an agent is not just concerned about the physical distance to its neighbors. Many other diverse and subtle aspects can be factored in, such as the maximum number of neighbors set by some cognitive limitations, familiarity and other social relationships, etc. The rule of *k*–nearest neighbors [63] epitomizes the topological paradigm. Figure 2 illustrates and highlights graphically some of the fundamental differences between a metric- and a topological-based neighborhood of interactions—the rule of *k*–nearest neighbors is considered. The metric neighborhood or interaction zone is an open ball with a constant radius, *R*, centered about the agent while *r*, the radius of the TNI, has an adaptive behavior to include the *k*-th (here 7-th) nearest neighbor. It is apparent that *r* is not just a function of the physical distance.

### Swarm signaling network

Let us consider members of a swarm, say a few hundreds, heading towards a certain destination. An individual agent lagging behind the large swarm, isolated from those moving together, decides to join the mainstream. Some information from the agents in the bulk of the swarm will flow towards the lonely agent and will almost surely affect its migratory behavior. Whereas agents within the swarm will most probably receive no information from the loner and will therefore experience no change in their behaviors. This phenomenon simply reflects the directed nature of the interactions among agents. Apart from this revealing case, empirical evidences support the idea of directed interactions in pigeon flocks [52].

We now precisely define and construct the SSN which, as already mentioned, is the information transfer channel underpinning the dynamics of the interacting swarming agents. Constituent links of the SSN of a group whose agents have directed interactions are unidirectional by opposition to bidirectional interactions in a group of agents with undirected interaction edges. The TNI based on the *k*-nearest neighbor rule allows one to locally identify the links between agents. The topological character of the neighborhood of interactions has a tremendous impact on the properties of interagent connectivity, in particular with the induced asymmetry in the relationship whereby if agent *j* is in the neighborhood of agent *i*, then *i* is not necessarily in the neighborhood of *j*, i.e. the interaction is directed. On the contrary, with a metric neighborhood the interagent connectivity is fundamentally symmetric with the presence of undirected interactions.

Through a bottom-up assembly of the interagent links, the complete global graph characterizing the connectivity can be constructed. Given the dynamics of the TNI and the directed nature of the links, the SSN is a switching strongly connected *k*–nearest neighbor digraph [30], [64], [65]. It is worth noting that the random graph theory [66]–[69] is not appropriate, nor relevant to the study of the dynamics of the connectivity in swarms since links are introduced irrespective of any distance between nodes—be that in the physical space or in the signaling network space.

### Dynamic swarming model

Above, we emphasized the generality of the concepts at the core of our modeling framework. Thus, details such as the nature of the state variables or the type of interactions between agents were intentionally left out. We believe that those specific details do not have an impact on the key features at the heart of emergence in collective behaviors; this approach can be regarded as a “crude look at the whole” as advocated by the Physics Nobel Laureate Murray Gell-Mann [70].

To exemplify our general framework for collective behaviors, we consider self-propelling agents moving about a two-dimensional plane with constant speed, 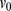, similarly to Vicsek's model [16]. However, our neighborhood of interactions is not metric but instead is topological. For simplicity, we assume that each agent 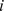 is fully characterized by one unique state variable 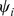, its velocity 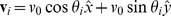, or equivalently its velocity direction 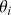, the speed 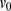 being constant. The local synchronization protocol, based on relative states and generically stated as in Eq. (21), is strictly equivalent to a local alignment rule which mathematically can be stated as:
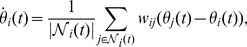
(23)where 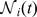 is the time-dependent set of outdegree neighbors in the TNI of agent *i*, with cardinal number 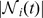, and 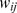 is the binary weight of the 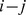 communication link. Note that in some models, 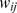 can take a more complicated form than our binary choice [20], [71], [72]. Using the 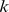-nearest neighbor rule for the TNI, we have 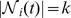 and the following dynamical equation for each individual agent in the swarm:
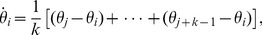
(24)where 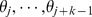 are its 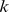-nearest neighbors' velocity directions. The dynamics of the agents in the two-dimensional physical space are intricately coupled to the dynamics of the SSN. This network is, by construction, a switching 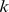-nearest neighbor digraph, for which the specific value of 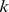 has a direct impact on its strongly connected character.

Up to this point, our modeling framework is based on a continuous-time approach. From a practical standpoint, it is necessary switching to a discrete-time approach; the associated sampling time, 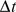, being intimately connected to some of the characteristic physical times of our complex dynamical system: e.g. agent's speed, speed of interagent information exchange, speed of internal information processing within one agent, etc. Once a sampling time 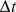 has been selected or is imposed by the natural or artificial characteristics of the system, the set of equations governing the discrete-time dynamics of the agents' property reads
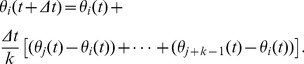
(25)It is worth highlighting here that the very fact that relative states are considered, prevents any singularity—such as those reported with the original Vicsek's model [73]—from occuring. As already mentioned, the formalism of Eq. (25) does not capture the fact that the value of the 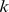 indices—from 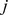 to 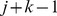 above—are actually 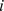-dependent since they are defined by the belonging, or not, of an agent to the TNI of agent 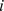. Moreover, these 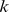 indices may change over time due to the dynamical nature of the TNI, itself imposed by the dynamics of agent 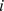.

The model devised here would not be realistic without accounting for the ubiquitous presence of noise which may have disruptive behavioral effects. This so-called behavioral noise can be divided into two broad categories: the stimulus noise and the response noise [55]. The stimulus noise, a.k.a. intensity noise, may have different origins like channel noise, environmental or background noise, and receptor noise. In the present framework, the channel, environmental and receptor noises are indistinguishable. In order to account for the global effects of stimulus noise together with external perturbing factors, a fixed level of background noise is considered throughout the agents' surroundings. In addition, the response noise may have different origins like motor noise and developmental noise which cannot be appropriately included within the present idealized modeling framework. In what follows, the response noise is therefore discarded and the stimulus noise may simply be referred to as noise without any possible confusion.

Noise can generally be assumed to be random fluctuations with a normal distribution [55]. In the sequel, the background noise is considered to have a normal distribution fully characterized by its noise level, 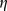. Specifically, the presence of noise modifies the equation governing the dynamics of agent 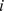 which now reads
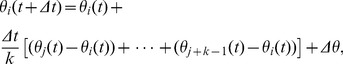
(26)where 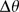 is a random number chosen with a uniform probability from the interval 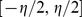.

### Simulation parameters

In all simulations, agents are distributed across a 25–by–25 square with periodic boundary conditions to avoid any boundary effect, while the time unit 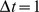 was the time interval between two updates of the directions 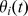 and the positions 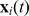 of each agent 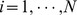. The synchronous position update is simply achieved through
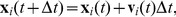
(27)where the velocity 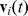 is calculated in its complex form 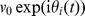 with the constant speed 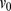 taken equal to 0.05. Similarly to Vicsek *et al.* [16], the value 0.05 for 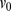 was chosen such that agents always interact with their neighbors and move fast enough to change the configuration after a few updates of the directions. According to our simulations, in a wide range of the speed (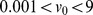), the actual value of 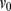 does not affect the results. In most of our simulations, for the initial conditions, agents are initially uniformly distributed in the two-dimensional spatial domain, with randomly distributed directions. Efficient ways of implementing such a swarm simulation code are discussed in Ref. [74]–[76].

The collaborative interactions of agents governs the dynamics of the self-organization of the swarm, ultimately leading (or not) to the emergence of consensus in the physical space. In the framework of our model, a good metric for the consensus in the physical space is given by the average alignment
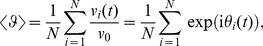
(28)over the 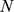 agents of the swarm; 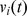 being the complex velocity of agent 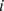 in the plane at instant 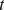. The alignment, 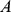, is defined by the absolute value of the steady-state average alignment: 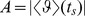, where 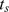 is the time required to reach a stationary state. This measure of the alignment approaches the unity if all agents in the swarm move more or less in the same direction, and is exactly equal to the unity if they are perfectly aligned. On the contrary, if the agents fail to reach consensus, the alignment will tend to zero, with the value 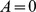 representing utter mess.
